# Experience in non-microscopic surgical management of complete penile amputation in a resource-limited setting: a case report

**DOI:** 10.1016/j.ijscr.2025.111427

**Published:** 2025-05-13

**Authors:** Tsedalu Worku Yifru, Seyfe Bekele Tilahun, Wondwossen Amtataw Zerefa

**Affiliations:** a Dolo Addo primary hospital, Somali region, Ethiopia; b Department of Surgery, Addis Ababa University, College of Health Sciences, Addis Ababa, Ethiopia; c Department of Surgery, Yekatit 12 Hospital Medical College, Addis Ababa, Ethiopia

**Keywords:** Case report, Penile amputation, Penile replantation, Microsurgery

## Abstract

**Introduction and importance:**

Penile amputation is a urologic emergency requiring prompt treatment to optimize outcomes. This report focuses on a case of self-inflicted penile amputation, its surgical management, and a review of current literature.

**Case presentation:**

A 40-year-old Ethiopian male with a known psychiatric illness arrived at the emergency department eight hours after a complete penile amputation. He underwent non-microsurgical replantation, reconnecting the corporal, fascial layers, and skin. Post-surgery, he recovered well with good erectile function, preserved sensation, and an acceptable appearance. He was discharged six weeks later.

**Clinical discussion:**

While microsurgical replantation is considered the gold standard, emerging evidence suggests that non-microsurgical techniques can achieve favorable outcomes, particularly when performed within a critical ischemia window. This case supports previous findings that penile replantation without microvascular anastomosis can still restore organ viability, adequate urinary function, and satisfactory sexual outcomes. The report underscores the potential role of corporal blood supply in maintaining penile perfusion even without vascular re-anastomosis.

**Conclusion:**

In cases of complete penile amputation without microsurgical tools, gross replantation is the preferred treatment, offering good functional and cosmetic outcomes.

## Highlights

• Non-microsurgical penile replantation achieved good functional and cosmetic outcomes.
• Successful replantation despite 10-hour ischemia in a resource-limited setting.
• Viability maintained without microvascular anastomosis, likely due to corporal blood supply.
• Postoperative recovery included wound healing, urinary function, and erectile restoration.
• Findings support non-microsurgical repair as an alternative when microsurgery is unavailable.

## 1 Introduction

Penile amputation is an infrequent emergency in the field of urology that needs to be addressed immediately to maximize functional outcomes [1]. Penile amputation is often due to self-mutilation during acute psychosis, but can also result from circumcision, violence, assault, or accidental trauma. Management has evolved from routine penectomy to organ reattachment using microvascular techniques [2]. The first penile replantation using microsurgical techniques was reported in 1929. The first successful reimplantations using microsurgical techniques, involving blood vessel and nerve re-anastomosis were also reported in 1977 [3,4]. However, there is currently no universally accepted regimen for the repair of penile amputation [5]. This case report examines the treatment and outcomes of non-microsurgical replantation of complete penile amputation and reviews relevant literature to highlight current clinical practices. This case is reported in line with the SCARE criteria [6].

## 2 Case presentation

**Fig. 1:**
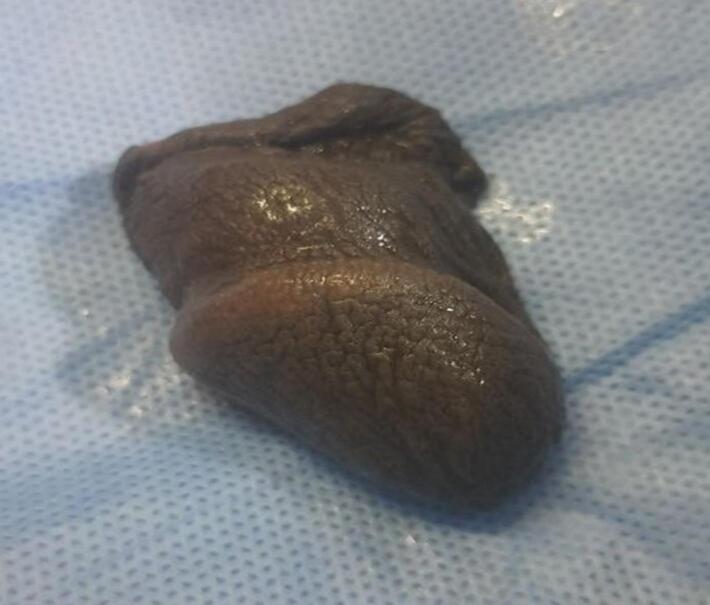
Amputated penis (distal part).

**Fig. 2:**
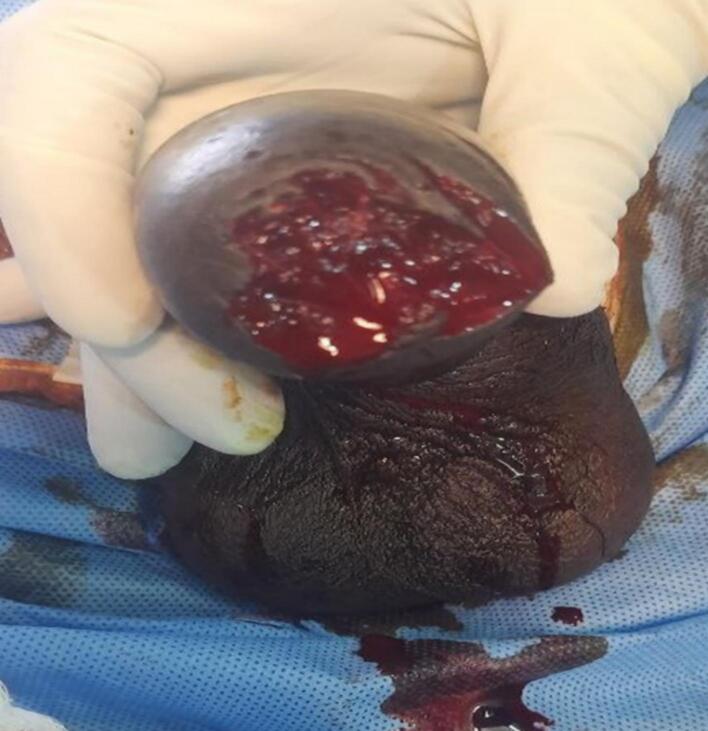
Amputated penis (proximal).

A 40-year-old unmarried Ethiopian male with a history of psychiatric illness arrived at a rural hospital eight hours after self-inflicting a complete penile amputation. The pre-operative assessment showed distal penile amputation with swelling, clotting, and blood oozing, while the testis and scrotum remained intact (Fig. 1, Fig. 2). The patient was resuscitated and given antibiotics, analgesics, and a tetanus shot. The wound was then packed, and the patient was taken to the operating room.

**Fig. 3:**
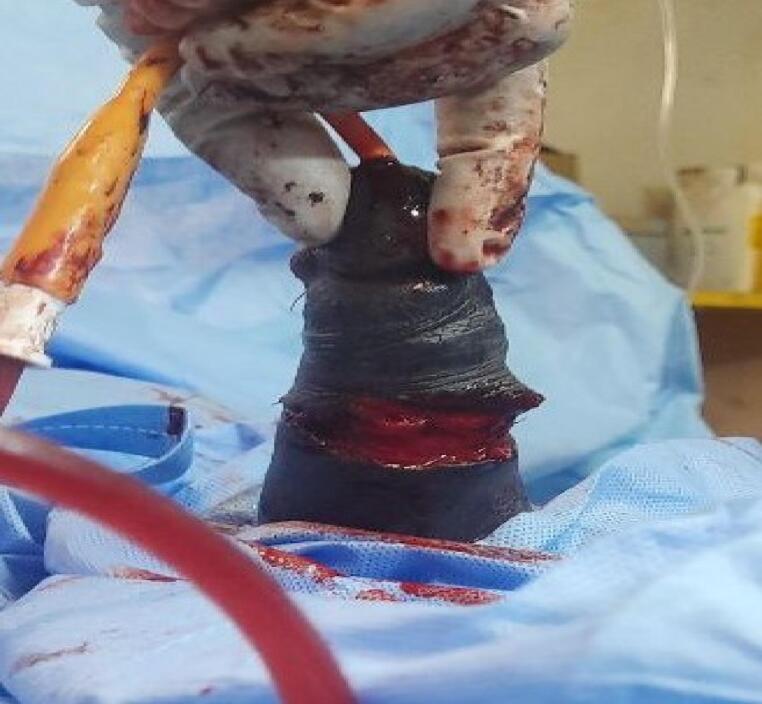
Intra-operative picture after urethra and erectile tissues are reconstructed.

**Fig. 4:**
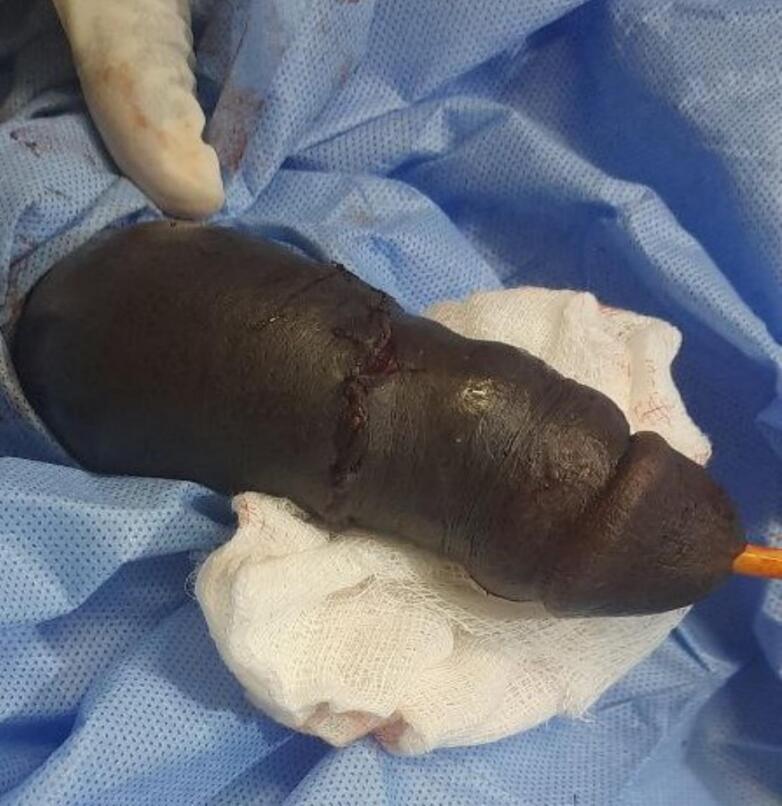
Intra-operative picture after all layers are reconstructed.

The patient underwent emergency surgery under spinal anesthesia. Saline irrigation was used to assess the wound, revealing a complete transection of the cavernosal bodies, spongiosum, urethra, veins, arteries, and nerves. In this patient with complete transection, we performed a primary end-to-end urethral anastomosis using eight interrupted 4–0 Vicryl sutures over a 16 Fr Foley catheter. The catheter was left for 3 weeks to facilitate adequate healing. This was followed by suturing the cavernosa and spongiosum. The fascial layer and skin were then closed, and pressure dressing was applied. The surgery was completed in 2 h, with a total ischemia time of 10 h (Fig. 3, Fig. 4).

**Fig. 5:**
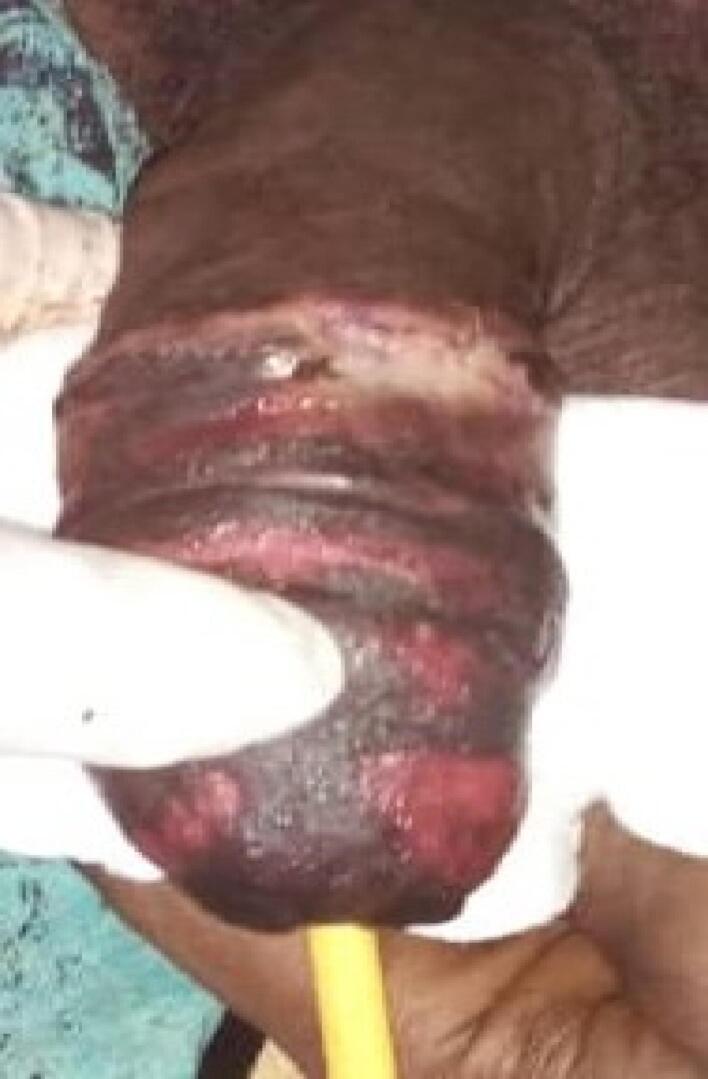
On the 14th post-operative day distal penile skin started to exfoliate.

The patient remained on bed rest for three days post-surgery, receiving IV ceftriaxone (2 g/day), tramadol (150 mg/day), and ketoconazole (400 mg TID) to reduce erections. A new sterile dressing was applied on the second day due to significant penile edema, which had subsided by the fifth day. By the seventh day, the epidermis of the distal penis was necrotized and exfoliated (Fig. 5).

**Fig. 6:**
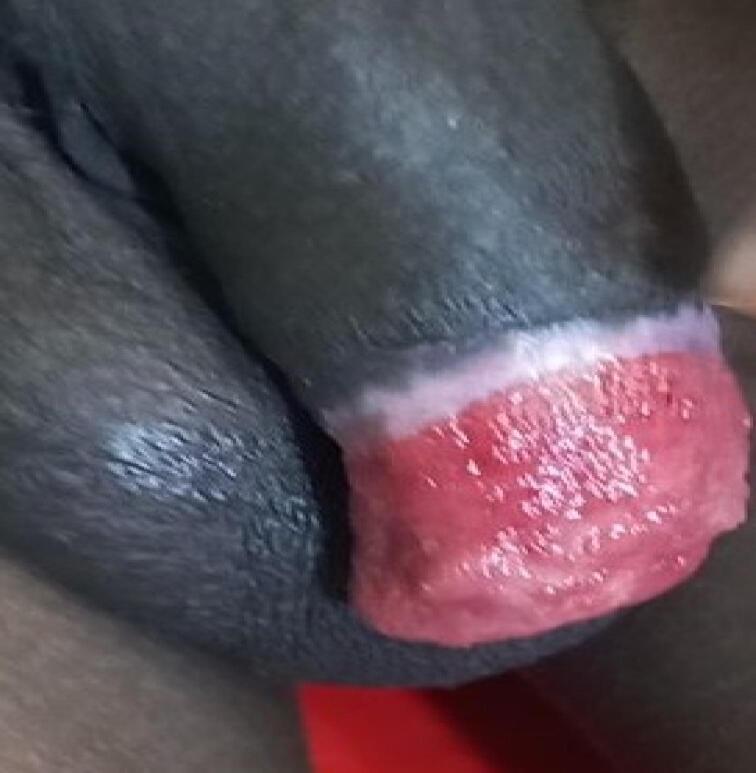
Distal Skin loss and granulating wound.

**Fig. 7:**
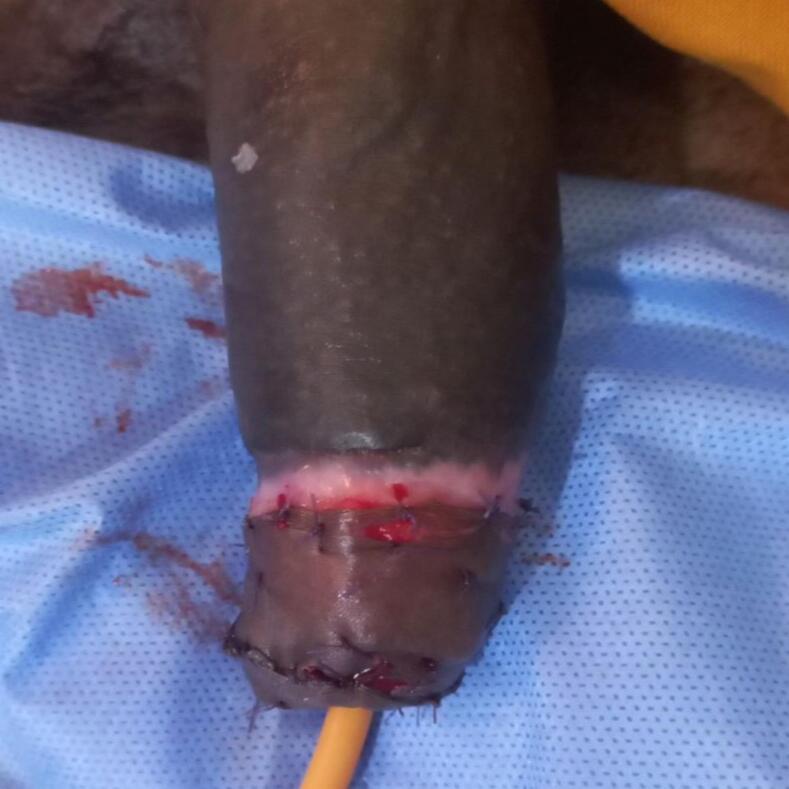
Split thickness skin graft.

**Fig. 8:**
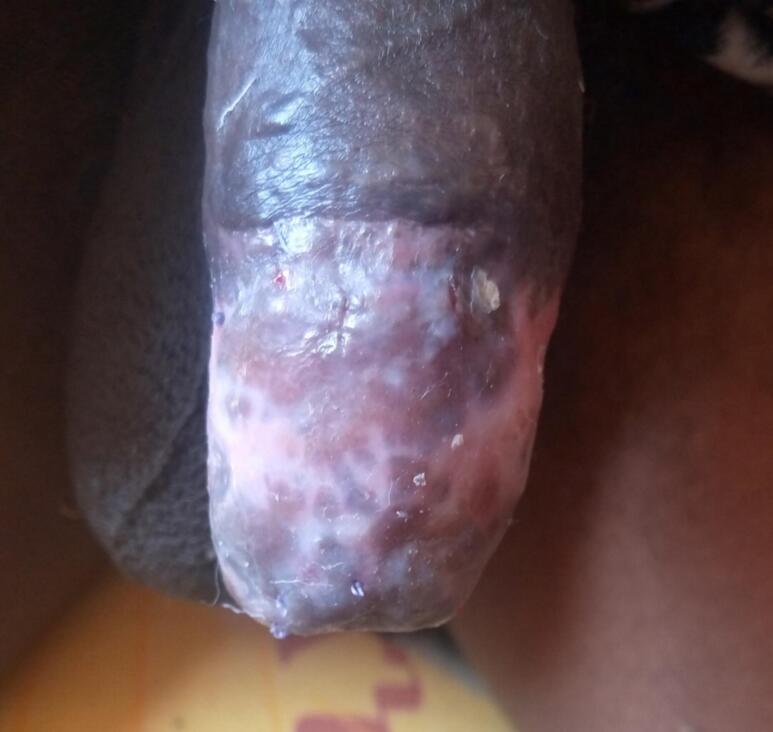
Three weeks after skin graft.

The patient received daily wound care, achieving good granulation by six weeks post-surgery (Fig. 6). A successful split-thickness skin graft, using donor skin from the medial thigh, was performed (Fig. 7). He was discharged and later evaluated with adequate wound healing, satisfactory erections, and an improved quality of life (Fig. 8).

## 3 Discussion

Genitourinary injuries account for 33–66 % of hospitalizations related to external genital trauma [7]. Due to anatomical differences, males are more prone to genital injuries, which are often caused by violence, accidents, or extreme exercise. Blunt injuries account for 80 % of genital trauma. Causes and classifications vary by age, location, and injury mechanism. In adults, self-mutilation, often linked to mental health issues, is a common cause of penile injury [7,8].

Penile amputation is a rare urologic emergency, with approximately 87 % of cases caused by self-mutilation due to psychiatric disorders, particularly Klingsor syndrome. This condition involves paranoid schizophrenia with command hallucinations. Injuries can vary from minor skin lacerations to complete amputation [8]. A minority of reported cases arise from accidental industrial trauma, masturbatory trauma, and assault by spouses [9].

The first penile reimplantation was reported using a non-microsurgical approach, which involved removing necrotic tissue, approximating structures, and applying a skin graft. After surgery, the patient developed a glans hematoma and, two years later, experienced urethral stricture and penile shortening, though the penis appeared normal [10]. The organ remained functional with minimal scarring. In 1977, Cohen successfully performed the first microsurgical penile replantation [5]. A systematic review of literature from 1966 to 2007 documented at least 30 successful penile replantations [5,11,12].

In 2017,106 penile replantation cases were reviewed, suggesting the procedure is safe and effective. In 2019 a review of 13 cases found that contamination or ischemia time didn't impact success unless the injury was severe. However, penile amputation remains a challenge due to limited cases and a lack of standardized techniques and protocols [5,12].

Penile replantation outcomes vary widely and are often based on subjective assessments by both the surgeon and the patient [11]. This includes organ survival, a good urinary stream, satisfactory cosmetic appearance, and the return of sensation and erections [2,5]. Several factors contribute to favorable outcomes, including ischemia duration, injury type and severity, mechanism of injury, and the use of a microscope during surgery [5,11].

Many studies suggest that the optimal window for successful replantation is within six hours post-amputation. However, good structural and functional recovery is reported even with ischemia times over 10 h [12]. Microsurgical repair after 16 h of cold ischemia or injuries lasting over 24 h has also shown promising results [13,14]. In this case, the patient's ischemia time exceeded eight hours (around ten hours), yet the final outcome demonstrated satisfactory functionality and cosmetic restoration.

Surgical reconstruction is often easier with penetrating trauma due to clearer identification of structures, while blunt trauma is more challenging due to deformed anatomy and unclear margins [[12], [13], [14], [15]]. In our case, the injury sustained was self-inflicted penile amputation.

Penile amputation can be classified as complete or incomplete based on the severity of the injury [16]. There is no clear definition of incomplete amputation. Liu et al. found that incomplete amputation with preserved vessels and nerves has a better prognosis than cases with neurovascular damage [12]. A retrospective analysis study found that total amputation, increased nerve coaptation, and anastomosis of the superficial dorsal artery were significantly associated with positive outcomes. They noted that complete penile amputation often results in better outcomes due to clearer access to neurovascular structures, though their data lacked detailed illustrations and vessel clarification [5].

The choice between microscopic and non-microscopic surgical techniques remains debated. However, microsurgical repair led to better physical and psychosocial outcomes. Early neurovascular anastomosis is crucial for a successful result [12]. Microsurgical techniques enable appropriate anastomosis or coaptation of structure, which allows better sensation and control of sexual function and leads to greater patient satisfaction. Jezior et al. reported that meticulous anastomosis of cavernosal arteries and dorsal structure was associated with erectile function [17]. A contemporary report recognized the role of microsurgical revascularization in maintaining early and adequate penile blood flow in order to achieve the best appearance and erectile and voiding function outcomes [11].

Given the characteristics of penile blood supply, favorable outcomes can be achieved even without re-anastomosing the blood vessels [16]. It has been suggested that the spongiosal bodies might contribute to arterial supply, venous drainage, and penile erection. Mensah et al. reported a successful non-microsurgical penile replantation with good voiding flow, cosmetic results, and erectile function, indicating that corporal bodies may assist in penile blood flow. From 100 cases reviewed with ischemia times up to 24 h found both microsurgical and microsurgical techniques to yield satisfactory outcomes, noting skin loss and urethra-cutaneous fistula as common complications. He recommended macrosurgical replantation if microsurgical resources are unavailable, as it still achieves good results [18].

Mendez et al. reported that non-microsurgical approaches resulted in necrotic skin, loss of penile sensation, urethral stricture, and urethrocutaneous fistulas [19]. In our case, we performed a non-microsurgical penile replantation for complete penile amputation using a gross re-implantation technique. The procedure resulted in distal skin loss which necessitated skin graft with resultant good postoperative outcomes and successful restoration without microsurgical methods.

In our case, the ischemic time was under eight hours, and reconstruction was completed in about two hours. While our case was managed using non-microscopic surgical techniques due to resource limitations, in general, magnification of around 6×–10× is commonly used in microsurgical repairs for larger structures such as the dorsal arteries, veins, and nerves. Despite not using a microscope for vessel anastomosis, the shorter ischemic time contributed to better overall outcomes. Additionally, distal penile injuries present a greater challenge for vascular anastomosis due to the involvement of smaller vessels [20].

## 4 Conclusion

Penile amputation is a rare urologic emergency with various causes. This case report discusses the treatment of self-inflicted penile amputation in a resource-limited setting. While the standard treatment is microscopic neurovascular reconstruction, non-microsurgical replantation in resource-limited settings can still produce satisfactory outcomes.

## CRediT authorship contribution statement

TWY was involved in conceptualization, data curation, writing original draft. SBT and AZ were involved in data curation and writing, reviewing and editing. All authors read and approved the final manuscript.

## Consent

Written informed consent is obtained from the patient for publication and any accompanying images. A copy of the written consent is available for review by the Editor-in-Chief of this journal on request.

## Ethical approval

Ethical clearance was obtained from the institutional review board (IRB) of the designated Hospital Medical College.

## Guarantor

Seyfe Bekele Tilahun.

## Funding

This research did not receive any specific grant from funding agencies in the public, commercial, or not-for-profit sectors.

## Declaration of competing interest

None.
